# Phytochemical Profiling of *Lavandula coronopifolia* Poir. Aerial Parts Extract and Its Larvicidal, Antibacterial, and Antibiofilm Activity Against *Pseudomonas aeruginosa*

**DOI:** 10.3390/molecules26061710

**Published:** 2021-03-19

**Authors:** Mahmoud Emam, Doaa R. Abdel-Haleem, Maha M. Salem, Lina Jamil M. Abdel-Hafez, Rasha R. Abdel Latif, Shaimaa Mahmoud Farag, Mansour Sobeh, Mohamed A. El Raey

**Affiliations:** 1 College of Pharmaceutical Science & Collaborative Innovation Center of Yangtze River Delta Region Green Pharmaceuticals, Zhejiang University of Technology, Hangzhou 310014, China; me.hegazy@nrc.sci.eg; 2 Phytochemistry and Plant Systematics Department, National Research Centre, Dokki, Giza 12622, Egypt; maha_abdelmejed@yahoo.com (M.M.S.); rasharefaat38@yahoo.com (R.R.A.L.); 3 Department of entomology, Faculty of Science, Ain Shams University, Abbasia, Cairo 11566, Egypt; doaaramadan@sci.asu.edu.eg (D.R.A.-H.); shaimaa.mahmoudfarag@sci.asu.edu.eg (S.M.F.); 4 Department of Microbiology and Immunology, Faculty of Pharmacy, October 6 University, 6th October City, Giza 12585, Egypt; linajamil@o6u.edu.eg; 5 AgroBioSciences Research Division, Mohammed VI Polytechnic University, Lot 660–Hay MoulayRachid, 43150 Ben-Guerir, Morocco

**Keywords:** *Lavandula coronopifolia*, *Culex pipiens*, larvicidal, antibiofilm formation, LC-MS/MS, molecular networking

## Abstract

Infections associated with the emergence of multidrug resistance and mosquito-borne diseases have resulted in serious crises associated with high mortality and left behind a huge socioeconomic burden. The chemical investigation of *Lavandula* *coronopifolia* aerial parts extract using HPLC–MS/MS led to the tentative identification of 46 compounds belonging to phenolic acids, flavonoids and their glycosides, and biflavonoids. The extract displayed larvicidal activity against *Culex pipiens* larvae (LC_50_ = 29.08 µg/mL at 72 h). It significantly inhibited cytochrome P-450 monooxygenase (CYP450), acetylcholinesterase (AChE), and carboxylesterase (CarE) enzymes with the comparable pattern to the control group, which could explain the mode of larvae toxification. The extract also inhibited the biofilm formation of *Pseudomonas aeruginosa* by 17–38% at different Minimum Inhibitory Concentrations (MICs) (0.5–0.125 mg/mL) while the activity was doubled when combined with ciprofloxacin (ratio = 1:1 *v*:*v*). In conclusion, the wild plant, *L.* *coronopifolia,* can be considered a promising natural source against resistant bacteria and infectious carriers.

## 1. Introduction

Mosquito vector-borne diseases are considered a global problem, which highlights the necessity for new prospects and cost-effective agents for vector control. Around 100 species of mosquitoes transmit viral and bacterial disorders such as malaria, lymphatic filariasis, dengue, and yellow fever, affecting several millions of people worldwide. In 2017, WHO recorded the highest mortality and morbidity due to mosquito-borne disorders that affect human health and economic society. Therefore, the development of novel mosquito repellents and antibacterial agents to overcome the microbial resistance threat is highly demanded. This could also help to avoid disrupting the ecological balance [1,2,3]. Plant secondary metabolites could furnish safe, efficacious, and multi-mechanistic candidates that might be useful as insecticidal and antibacterial agents.

The genus *Lavandula* (commonly known as lavender) comprises 45 species that are mainly distributed in subtropical and tropical regions [4,5]. Plants of the genus have been used in folk medicine since ancient times to treat pain, headache, migraine, and antiepileptic, antidiuretic, antirheumatic, and carminative agents. They become famous for their multiple uses in different pharmaceuticals, aroma, and food products [6,7]. The phytochemistry of the genus is centered on mono- and sesquiterpenoids, together with traces of alkaloids, and phenolic structures [7].

*Lavandula coronopifolia* Poir. (Arabic: Khozama) is a shrublike perennial, growing in the rocky environment and desert plains mainly distributed in subtropical and tropical regions [8]. The first attention to *L. coronopifolia* traces back to 1999, when El-Garf et al. isolated and identified numerous hydroxyl flavones such as hypolaetin, isoscutellarien, and luteolin from its dried aerial parts [4]. Then, several studies reported the presence of polyhydroxyoleanolic acids, polyhydroxyursolic acids and their glycosides, caffeic acid, rosmarinic acid, rutin, quercetin, and hesperidin [9,10].

*L. coronopifolia* showed a plethora of substantial biological activities. These include antioxidant [11], antimicrobial [12], α-glucosidase inhibitory [10], and hepatoprotective [13] activities. These activities were attributed to the presence of flavonoids, especially flavones and their glucuronides, in addition to triterpenes [9,10]. Moreover, *L. coronopifolia* essential oils possessed substantial antibacterial activity against the Gram-negative bacteria and methicillin-resistant *Staphylococcus aureus* bacteria [14].

In this work, we comprehensively characterized the phytoconstituents of the aerial parts of *L. coronopifolia* extract by HPLC–MS/MS and confirmed the existence of possible skeletons quantitatively using ^1^H-NMR and molecular networking. We also evaluated the insecticidal activities against *Culex pipiens* larvae and the antibiofilm formation activity against isolates of *Pseudomonas aeruginosa*. We explored several biochemical parameters to investigate the mechanism of the insecticidal activities.

## 2. Material and Methods

### 2.1. Plant Material, Extraction, and Preliminary Qualitative Analysis

*Lavandula coronopifolia* Poir. was collected from the Western Desert of Egypt in March 2018. A voucher sample was placed at the international herbarium of the National Research Centre (CAIRC) (S.N: 1023). The plant aerial parts (250 g) were crushed into small pieces and then extracted by dipping into 70% MeOH:H_2_O (*v:v*) at ambient temperature for one week. The solution was filtered (Whatman no. 1), then concentrated till dryness using Rotavapor^®^ (Heizbad Hei-VAP, Heidolph, Germany), yielding 28.45 g, and stored at 4 °C for further experiments. The *L. coronopifolia* extract was screened for its phyto-constituents as flavonoids (Shinoda’s test), phenolics (FeCl_3_ test), ellagitannins (NaNO_2_ assay), and gallotannins (KIO_3_ test) [15,16]. The proton NMR (Jeol ECA-500 MHz, Japan) experiment using DMSO-*d6* was done for the total extract.

### 2.2. HPLC-MS/MS

HPLC-PDA-MS^n^ mass spectra was performed through a ThermoFinnigan (Thermo Electron Corporation, Austin, TX, USA) LC system coupled with a mass spectrometer (LCQ-Duo ion trap) having an ESI source (ThermoQuest, Thermo Scientific, Waltham, MA, USA) [15]. The injection process, flow rate, elution solvents, resolution, and negative MS operating parameters were described previously [17]. In brief, a Zorbax Eclipse XDB-C18, rapid resolution, 150 × 4.6 mm, 3.5 μm column was used (Agilent, Santa Clara, CA, USA). A gradient consisting of water and acetonitrile (ACN), each having 0.1% formic acid, was applied, and ACN was increased from 5% to 30% within 60 min and then to 90% within the next 30 min at a flow rate of 1 mL/min and a 1:1 split before the ESI source [17].

### 2.3. Molecular Networking Workflow Description

The mgf formatting mass file was uploaded to the online platform of GNPS (http://gnps.ucsd.edu) (accessed on 27 June 2020). Then the data were filtered as described previously and the visualization of molecular networking (MNW) workflow was carried out using Cytoscape 3.6.1 software [15,18].

### 2.4. Larvicidal Assay

#### 2.4.1. Insects

A laboratory susceptible strain of *Culex pipiens* was obtained from the Research and Training Center on Vectors of Diseases (RTC), Ain Shams University. It was colonized in the entomology department insectary at 27 ± 2 °C, 75 ± 5% relative humidity (RH), and a 14 h/10 h light/dark photoperiod following standard procedures [19]. The larvae were reared in enamel dishes containing 2000 mL of distilled water. Newly hatched larvae were fed on Tetra-Min, Germany. Adults were reared in (30 × 30 × 30 cm) wooden cages and provided with 10% sucrose solution daily, as well as a pigeon for female blood feeding.

#### 2.4.2. Bioassay

The larvicidal activity was evaluated against the third larval instar of *C. pipiens* under the same controlled laboratory conditions. The bioassay was assessed using the standard method described in [20]. The extract was dissolved in water to prepare the stock solution. Batches of 25 of 3rd instar larvae of *C. pipiens* were transferred by a plastic dropper to small disposable test cups and treated with different concentrations of the extract (10, 25, 50, 100, 150, and 200 µg/mL prepared in distillated water) and control with distillated water only, in a triplicate manner. Mortality was recorded after 24, 48, and 72 h post treatment.

#### 2.4.3. Preparation of Samples for Biochemical Assay

The 3rd larval instar of *C. pipiens* was treated by LC_50_ values, and then the insects were prepared as described by Amin et al. [21]. The whole bodies of larvae were homogenized in distilled water (50 mg/1 mL). The homogenates were centrifuged at 8000 r.p.m. for 15 min at 4 °C. The supernatants were used for biochemical analyses. Acetylcholinesterase (AChE) and carboxylesterase assays were measured according to the method described by Simpson et al. [22], using acetylcholine bromide (AchBr) and methyl n butyrate (MeB) as substrates, respectively. Alpha esterase (α-esterase) activity was determined according to Van Asperen [23] using α-naphthyl acetate as substrate. Glutathione S-transferase (GST) catalyzes the conjugation of reduced glutathione (GSH) with 1-chloro 2,4-dinitrobenzene (CDNB) via the –SH group of glutathione. The conjugate S-(2,4-dinitro-phenyl)-l-glutathione could be detected as described by the method of Habig et al. [24]. Cytochrome P-450 monooxygenase activity was determined using *p*-nitroanisole o-demthylation according to the method of Hansen and Hodgson [25] with slight modifications.

### 2.5. Microbiological Assay

#### 2.5.1. Sample Collection and Identification of Isolated Bacteria

Clinical isolates of *Pseudomonas aeruginosa* were collected from burn wounds, otitis media, and urine as previously described [26] and were kept for scientific research. The isolates were grown on Tryptic soya agar (TSA) (Difco^TM^, Strasbourg, France) for 24 h at 37 °C, then one single colony of each isolate was inoculated into 2 mL Tryptic soya broth (TSB) (Difco^TM^, France) with overnight incubation at 37 °C. The samples were cultivated on Cetrimide agar media, after which the isolated species were identified by morphology (pale yellow colonies on MacConkey and green exopigment on Cetrimide agar), Gram staining (Gram-negative bacilli), and biochemical reactions (oxidase-positive). The Microbact™ Gram-negative system was implemented in compliance with the manufacturer’s protocol (Oxoid, Hampshire, UK). A standard strain of *P. aeruginosa* (ATCC 12924) was kindly provided by NAMRU as a frozen cultural broth containing 40% glycerol.

#### 2.5.2. The Antimicrobial Susceptibility Testing

The antimicrobial susceptibility testing was done using cup agar diffusion method [27]. The bacterial cultures were adjusted to an optical density (OD) of 0.5 at 600 nm, TSA plates were covered with 100 µL of each bacterial isolate, and 5 mm pores were filled with 100 µL of extract dissolved in sterile water at 2.5 mg/mL. The plates were incubated for 24 h at 37 °C. The zone of inhibitions was measured in mm.

#### 2.5.3. Minimum Inhibitory Concentration (MIC)

MIC is used as the gold standard method for detecting the sensitivity of the organisms to antimicrobial agents [28]. The final concentrations of the extract ranging from 2.5 to 0.0195 mg/mL were prepared and then added to test tubes containing 1 mL of sterile TSB media. The bacterial suspensions with OD 0.5 at 600 nm were diluted 1:100 (≈106 CFU/mL), and then 50 μL of inoculums were added to each tube. The tubes were incubated at 37 °C ± 2 °C for 24 h. A tube containing TSB broth without extract was taken as control. The MIC was defined as the lowest concentration of the tested extract that restricted the visible growth of tested strains compared to the blank [29].

#### 2.5.4. Minimal Bactericidal Concentration (MBC)

The minimal bactericidal concentration (MBC) was determined by the Petri dish sowing method [30]. This procedure was dependent on the procedures for the determination of MIC. After the incubation period for the determination of the MIC, an aliquot of 0.1 mL was taken from each of the test tubes that were not showing growth, and then was inoculated into a TSA agar plate. The plates were then incubated at a temperature of 37 ± 2 °C for 24 h. After this period, the presence of bacterial colonies was observed in each plate. The MBC was defined as the lowest concentration of the plant extract that was able to prevent microbial growth in a culture medium (formation of bacterial colonies). The bioassays were performed in duplicate with three repetitions for each bacterial isolate.

#### 2.5.5. Biofilm Formation Assay and Quantification

The biofilms were assayed as described in [31,32] using sterile 96-well microtiter plates, each well containing 180 μL TSB broth and 20 μL of bacterial suspension with OD 0.5 at 600 nm. After 24 h incubation at appropriate conditions, all the planktonic cells were removed, and the biofilms were gently washed twice with phosphate buffer saline (PBS) to remove any free-floating bacteria. The biofilm cells formed in each well were stained with 200 μL crystal violet (0.1% *w*/*v*) and incubated at room temperature (28 °C) for 10 min. The stain was removed and washed with distilled water for 30–60 s. After 5 min of air drying, the biofilms were solubilized by 200 μL of 98% ethanol, then the optical densities of stained adherent biofilms were measured at 620 nm using a microplate reader. The evaluation of biofilm production was categorized according to the criteria of Stepanović et al. as follows: OD ≤ ODc: not a biofilm producer (non-adherent); ODc < OD ≤ 2ODc: a weak biofilm producer (weakly adherent); 2ODc < OD ≤ 4ODc: a moderate biofilm producer (moderately adherent); 4ODc < OD: a strong biofilm producer (strongly adherent). ODc and OD were defined as the mean OD of the blank wells and wells with biofilm, respectively [33].

#### 2.5.6. Biofilm Inhibition Assay

The ability of the extract to inhibit the biofilms of the clinical isolates of *P. aeruginosa* was evaluated according to Stepanović et al. [34] with some modifications. Microbial biofilms were developed in a round-bottom 96-well microtiter plate. Each clinical isolate was inoculated into each well of the 96-well microtiter plate. The extract was added to each well at 1/2, 1/4, and 1/8 MICs and incubated for 24 h at 37 °C. After the incubation period, non-adherent cells were detached by dipping each sample three times in sterile PBS. The samples were fixed for one hour, and the biofilms were stained with 0.1% solution of crystal violet in H_2_O. After staining, the samples were washed with distilled H_2_O (DW). The measurable biofilm production was achieved by adding 125 µL of 30% acetic acid to de-stain the samples. Afterwards, the OD at 620 nm was detected using the microplate reader. The percentage (%) of inhibition formula is as follows:

$$\%\mathrm{Inhibition}\;=\frac{\mathrm{Abs}\;\mathrm{control}\;-\mathrm{Abs}\;\mathrm{sample}}{\mathrm{Abs}\;\mathrm{control}}\times \;100$$

#### 2.5.7. Combination of the Extract with Ciprofloxacin

The ability of ciprofloxacin/extract (1:1) to inhibit the biofilm of the strong biofilm isolate of *P. aeruginosa* was evaluated according to Stepanović et al. [34] with some modifications. Microbial biofilms were developed in a round-bottom 96-well microtiter plate. The clinical isolate C4 was inoculated into each well of the 96-well microtiter plate, and ciprofloxacin/extract (1:1) was added to each well at 1/2, 1/4, and 1/8 MICs and incubated for 24 h at 37 °C. After incubation (24 h), non-adherent cells were detached by dipping each sample three times in sterile PBS. The samples were fixed for 1 h, and the biofilms were stained with 0.1% solution of crystal violet in H_2_O. After staining, the samples were washed with DW (distilled H_2_O). The quantitative analysis of biofilm production was achieved by adding 125 µL of 30% acetic acid to de-stain the samples. Afterwards, the OD at 620 nm was measured using the microplate reader. The percentage (%) of inhibition formula is as follows:

$$\%\mathrm{Inhibition}\;=\frac{\mathrm{Abs}\;\mathrm{control}\;-\mathrm{Abs}\;\mathrm{sample}}{\mathrm{Abs}\;\mathrm{control}}\times \;100$$

### 2.6. Statistical Analysis

The biofilm formation inhibiting activities of different concentrations of the extract were compared by two-way ANOVA (Bonferroni post hoc tests). *p* < 0.05 was used to detect the significance of differences. The obtained larvicidal data were analyzed using a statistics package (LDP-line) for goodness of fit (chi square test) and to detect LC_50_ and LC_95_ values with corresponding 95% confidence limits (CL), slope, correlation coefficient and standard error. The results of biochemical determinations were investigated by one-way analysis of variance (ANOVA) using Costat statistical software (Cohort software, Berkeley). When the ANOVA statistics were significant (*p* < 0.01), the means were compared by the Duncan’s multiple range test [35]. GraphPad Prism 5.0 software (GraphPad Prism Software Inc., San Diego, CA, USA) was used to draw most of the figures.

## 3. Results and Discussion

### 3.1. Phytochemical Screening and LC-MS/MS Profile of L. coronopifolia

**Figure 1:**
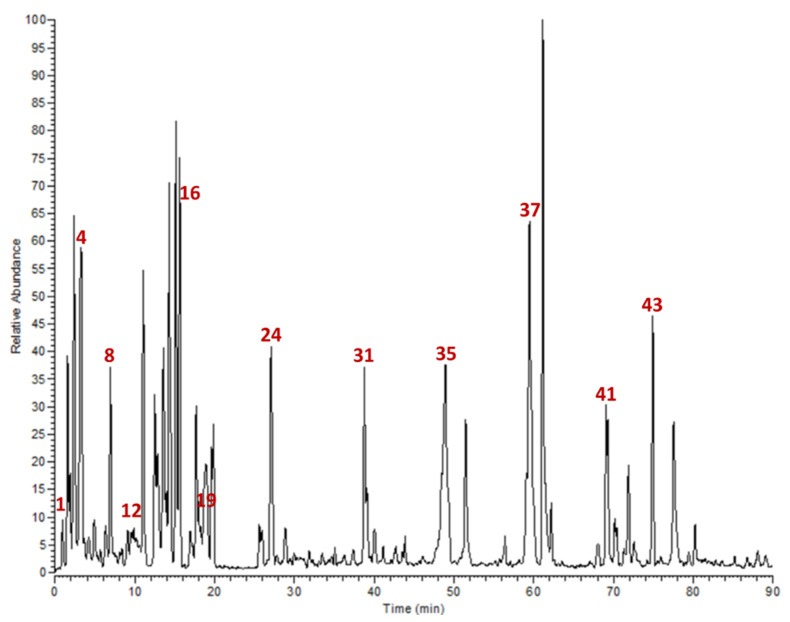
Base peak chromatogram of *L. coronopifolia* Poir. aerial parts’ extract.

The preliminary phytochemical screening of *L. coronopifolia* extract revealed the presence of dihydroxy phenolics and flavonoids and/or their glycosides, as well as the absence of ellagic and gallotannins moieties. The mass spectrometry analysis (full scan and product ion scan mode) provided structural information of 46 metabolites, including organic and phenolic acids, flavonoids and their glycosides, and bioflavonoids (Table 1 and Figure 1).

### 3.2. Molecular Networking (MNW) of L. coronopifolia Aerial Parts’ Metabolite Perception

**Figure 2:**
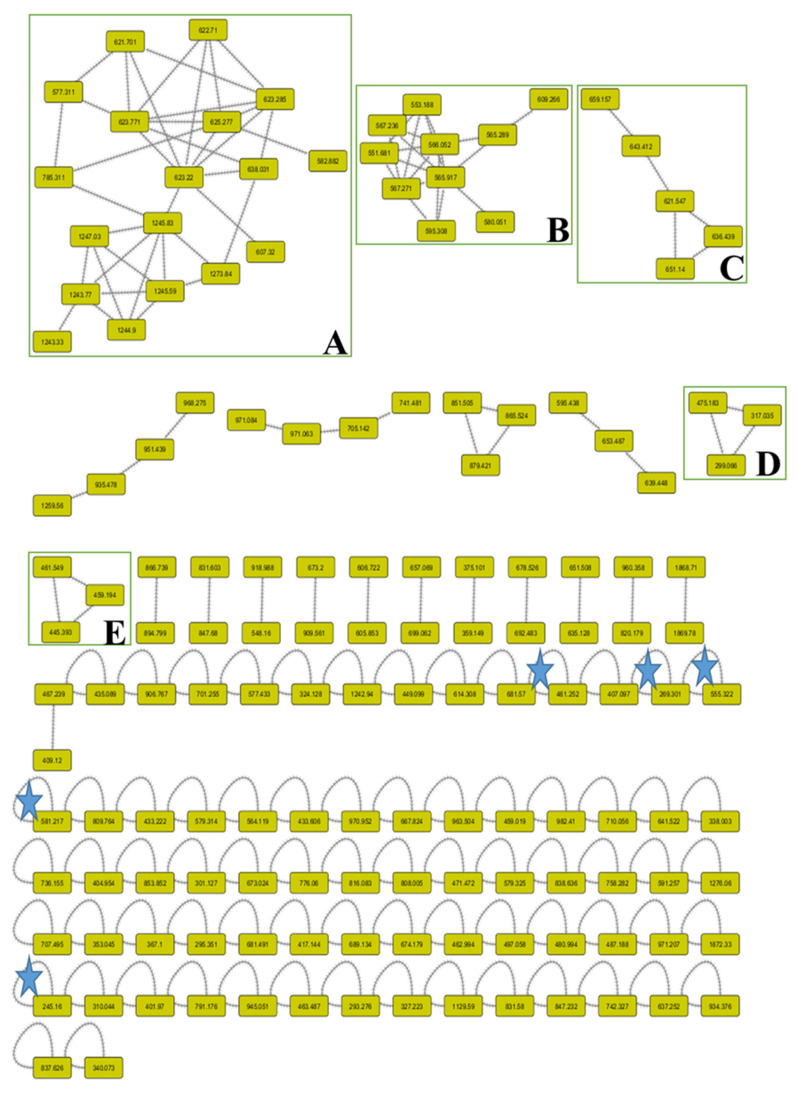
Complete molecular networking (MNW) generated using MS/MS data in (–ve) negative mode from *L. coronopifolia* aerial parts’ extract. Nodes are labeled with parent mass.

The symmetrical chemical entities were facilitated to be visualized through the molecular networking between the identical fragments (*m/z*) of definite metabolites. Through the network, each node was characterized with the parent mass [M–H]^−^ and the contiguous arrows (edges) connected between the similar nodes. The network was built for the negative ionization (–ve) mode using the GNPS 2 platform (Figure 2). The (–ve) network involved 148 nodes, 71 self-looped (individual) nodes, and 92 connected components. The designed networks facilitated the visual examination of the different compound families and analogues and assisted in isomer differentiation.

In the negative network, five clusters, A, B, C, D, and E, were mentioned and annotated as apigenin derivatives, which were grouped as methylated flavones, *O*-glycosidic flavones, *C*-glycosidic flavones, and/or biflavones. In addition, the other self-looped nodes asterisked within the network were denoted as phenolic-*O*-glycosides, N-acetylamino acid, flavan-3-ol, phenolic acids, and biflavones.

**Organic acids:** Quinic acid was determined with an [M-H]^–^ ion at *m/z* 191 fragmented to *m/z* 111, and 173, whereas malic acid was characterized with an [M-H]^–^ ion at *m/z* 133 and fragmented to *m/z* 115, 89, and 71. **Phenolic acids:** These structures were tentatively identified as eucomic acid, syringic acid-4-*O*- hexoside, sinapic acid 3-*O*-glucoside, and dihydrosinapic acid hexoside. They showed negative molecular ions at *m/z* 239, 359, 375, 385, and 387, respectively. These structures were tentatively identified depending on the main fragments that are shown in Table 1. **Flavonoids and their glycosides:** Most of the aglycones were found to be apigenin derivatives and their *O*- and/or *C*-linkage of mono and/or diglycosides. The MS of *C*-glycosides was characterized through the main fragmentations by the loss of different masses of 60, 90, 120, and 240 Daltons [36]. A molecular ion [M-H]^–^ at *m/z* 289 and yielding a main fragment at *m/z* 245 was identified as catechin. **Biflavonoids:** Several signals that gave parent ions [M–H]^−^ of *m/z* 551, 555, 565, 579, 581, and 609 were fragmented into specific fragments that characterized bioflavonoid derivatives [37]. Their identification, retention times, molecular weights, and fragmentation pattern are shown in Table 1.

**Table 1:** Chemical composition of *L. coronopifolia* Poir. aerial parts’ extract using LC-MS/MS.

No.	*t*_R_ (min)	[M–H]^−^	MS^2^ (*m/z*)	Tentatively Identified Compound	Ref.
1	1.68	191	111, 173	Quinic acid ^a^	[38]
2	1.75	133	89, 71, 115	Malic acid ^a^	[39]
3	3.51	211	137, 179	Caffeic acid derivative ^b^	
4	4.03	239	149, 179, 221	Eucomic acid ^b^	
5	4.34	359	315, 197, 153	Syringic acid 4-*O*-hexoside ^b^	[38]
6	4.61	237	115, 121, 137	2-(4-hydroxybenzyl)-malic acid ^b^	
7	5.03	375	125, 169, 213	Sinapoyl trihydroxybenzoic acid ^b^	
8	6.31	475	197, 359	Rosmarinic acid malate ^b^	
9	7.78	461	153, 315	Protocatechuic acid rhamnosyl glucoside ^b^	
10	8.95	245	203, 186, 115	*N*-Acetyltryptophan ^c^	
11	9.18	289	245	Catechin ^d^	[40]
12	9.29	299	115, 133, 183	Methyl trihydroxybenzoic acid malate ^b^	
13	13.54	385	223, 179	Sinapic acid 3-*O*-glucoside ^e^	
14	13.69	387	433, 225, 179	Dihydrosinapic acid hexoside ^e^	
15	14.51	387	433, 225, 179	Dihydrosinapic acid hexoside ^e^	
16	15.61	387	433, 225, 179	Dihydrosinapic acid hexoside ^e^	
17	17.57	389	227	Resveratrol glucoside ^f^	
18	17.72	461	285	Isoscutellarein-8-*O*-glucuronide **^#,^**^g^	
19	18.25	359	197, 179, 161, 135	Rosmarinic acid ^b^	
20	18.77	389	227	Resveratrol glucoside ^f^	
21	19.88	461	285	Luteolin-7-*O*-hexouronide ^g^	[6]
22	20.67	593	503, 473, 383, 353	Apigenin di-*C*-hexoside ^g^	[6]
23	25.79	445	269, 175	Apigenin-7-*O*-hexournide I ^@,g^	[6]
24	27.05	445	269, 175	Apigenin-7-*O*-hexouronide II ^g^	[6]
25	28.62	623	477, 461, 315	Hypolaetin 4′-*O*-methyl ether-*O*-hexoside-*O*-rhamnoside I ^g^	
26	33.57	623	477, 461, 315	Hypolaetin-4′-*O*-methyl ether-*O*-hexoside-*O*-rhamnoside II ^g^	
27	32.30	623	179, 315, 461	Isorhamnetin *O*-hexoside-*O*-rhamnoside ^g^	
28	34.98	607	461, 315, 299	Hypolaetin di-*O*-rhamnoside ^g^	
29	35.08	447	285	Luteolin-7-*O*-glucoside **^#,^**^g^	[6]
30	37.23	637	491, 461, 315	Hypolaetin 4′-*O*-methyl ether-8-glucuronide-O-rhamnoside ^g^	
31	38.80	459	283, 268	Acacetin-*O*-hexouronic acid ^g^	[41]
32	41.04	577	269	Apigenin-*O*-caffeoyl rhamnoside ^g^	
33	41.14	461	299, 283	Methoxy leteolin-7-*O*-hexoside ^g^	
34	43.90	651	505, 475, 329	Tricin-*O*-feruloyl rhamnoside ^g^	
35	48.62	621	459, 313	Crismaritin-*O*-caffeoyl rhamnoside ^g^	
36	51.41	327	171, 229, 327	Unknown	
37	59.10	313	298, 284, 269	Luteolin-7,3′-dimethyl ether ^g^	[6]
38	61.18	269	269, 151, 149	Apigenin ^g^	[6]
39	62.63	555	403, 429, 327, 299	Binaringenin methyl ether ^h^	[37]
40	69.07	551	457, 431, 389	Methoxy amentoflavone ^h^	
41	69.42	553	458, 432, 390	Dihydrobilobetin ^h^	
42	71.92	283	268, 133	Acacetin ^g^	[41]
43	75.90	565	471, 389	Dimethoxy amentoflavone ^h^	
44	78.77	609	577, 551, 489, 269	Penta methoxy dihydro biapigenin ^h^	
45	81.37	579	533, 485, 389, 268	Kayaflavone ^h^	
46	81.81	581	579, 535, 487	Dihydrokayaflavone ^h^	

^#^ Isolated before from the same species [4]. **^@^**Confirmed by UV *λ*_max_. ^a^ Organic acid, ^b^ phenolic acid, ^c^ amino acid derivative, ^d^ flavan-3-ol, ^e^ phenylpropanoic acid, ^f^ stilbenoid, ^g^ flavone, and ^h^ biflavonoid.

### 3.3. ^1^H-NMR Analysis of L. coronopifolia Extract

The extract was dissolved in deuterated dimethyl sulfoxide (DMSO-*d6*) and introduced into the proton NMR (500 MHz) experiment. The resonated peaks at different chemical shifts explained the kind of protons present in the chemical structures and represented the classes of skeletons in the solution. The 2-phenyl chromen-4-one of the flavone structure bearing substituents at positions 5 and 7 of A-ring was observed as the main skeleton. Through the ^1^HNMR chart, the downfield protons that appeared as a doublet at δ 7.93 ppm were characterized for H-2′ and 6′ of B-ring, while the protons resonating at δ 6.92 ppm were assigned for H-3′ and 5′ of B-ring, with the *ortho*-coupling constant (*J* = 9.0 Hz) suggesting that the B-ring bearing the hydroxyl group was at position 4′, while the appearance of a singlet proton at δ 6.83 ppm was characterized for H-3 of the flavone structure. In addition, the downfield shift observed at δ 6.42 and 6.81 ppm for H-6 and H-8 of the A-ring of the flavone structure with *meta*-coupling (2 Hz) or broad singlet was predicted due to the substitution at position 7. The previous interpretation confirmed the presence of the apigenin derivative in the solution with substitution at position 7. In addition, the ABX system of B-ring was observed along with the chart through the coupling constant of resonated protons as a doublet of doublet (*ortho*- and *meta*-coupled), doublet (*meta-*coupled), and doublet (*ortho*-coupled) at δ 7.46, 7.43, and 6.92 ppm, respectively, were assigned, respectively, for H-6′, H-2′, and H-5′ of the B-ring, suggesting that the B-ring was substituted with substituents at positions 3′ and 4′. The rest of H-3, H-6, and H-8 proton signals resonated around the mentioned chemical shifts, which confirmed the presence of a luteolin derivative with 7-*O*-substituents. In addition, the resonated singlet signal at δ of 3.83 ppm was assigned to 4’-O-methylated flavone (i.e., the presence of an acacetin or methyl apigenin derivative in the solution). Moreover, the series of signals between δ 3.14 and 3.51 ppm were attributable to a sugar moiety, and the doublet signal with a coupling constant of 7.2 Hz was distinctive for the anomeric proton of sugar with O-*β*-d-linkage. Depending on the previously published data and MS assignment, the major attached sugar for the flavone structure is glucuronide moiety.

### 3.4. Insecticidal Activity

Plant extracts are considered a new ecofriendly and efficient alternative means for controlling mosquitoes. The larvicidal activity of the extract was evaluated against the 3rd instar larvae of *C. pipiens.* The fiducial limits were calculated for LC_25_, LC_50_, and LC_95_ at *p* < 0.05 (Table 2). The extract exhibited considerable larvicidal activity against *C. pipiens* larvae where the LC_50_ values after 24, 48, and 72 h of exposure were 52.74, 34.07, and 29.076 µg/mL, respectively. The essential oil from the Egyptian plants exhibited insecticidal activity against the 4th larval instar of *C. pipiens* [42]. Similar activities were reported from essential oils of other *Lavandula* species, among them *L. stoechas* and *L. dentata* [43,44].

### 3.5. Biochemical Activity

**Figure 3:**
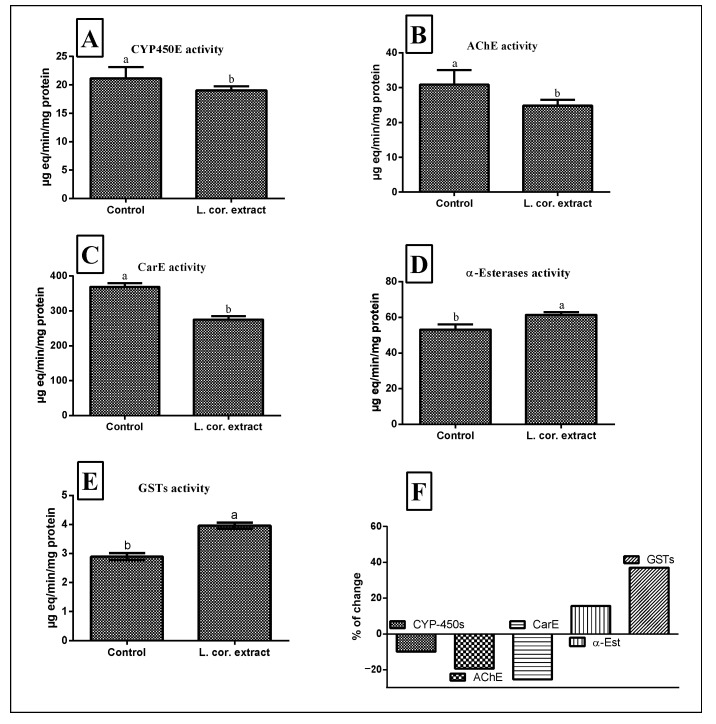
Effects of *L. coronopifolia* extract (L. cor.) on the enzymatic activities of cytochrome P-450 monooxygenase (CYP-450s) (**A**), carboxylesterase (CarE) (**B**), acetylcholinesterase (AchE) (**C**), α-esterases (**D**), and glutathione S-transferase (GSTs) (**E**) in the 3rd larval instar of *C. Pipiens*. (**F**); % of changes. Data were represented as mean ± SE. Lowercase letters above the bars indicate significant differences between different treatment groups (Duncan’s multiple range test, *p* < 0.01). Error bars indicate 95% confidence intervals.

Insects release several detoxifying enzymes such as esterases, oxidases, and reductases to face and detoxify many invader pesticides [45]. To get an insight into the mechanisms involved, we explored the activities of five different enzymes in the 3rd larval instar of *C. pipiens*. The extract, at a concentration of LC_50_ and exposure time of 72 h, inhibited cytochrome P-450 monooxygenase, acetylcholinesterase, and carboxylesterase by −9.92%, −19.41%, and −25.47%, respectively, compared to the control group (Figure 3), while the treated larvae showed elevation of α-esterases and glutathione S-transferase contents by 15.63% and 37.02% compared to the untreated larvae. This could highlight the significant role of glutathione S-transferase and α-esterases in the detoxification mechanism of the extract. Our results come in agreement with those of the Huang et al. study, which reported an increase in the intracellular glutathione content when the larvae were treated with a polyphenolic-rich extract [46,47,48].

As the time of exposure increased, the toxicity of the extract to the 3rd instar larvae increased, followed by a substantial decrease in carboxylesterase (CarE), acetylcholinesterase (AChE), and cytochrome P-450 monooxygenase (CYP450) levels. This suggests the temporary response and neurotoxic effects of the extract. Our results come in agreement with those of the Gershenzon et al. and Salunke et al. studies, which reported the inhibitory effect of natural secondary metabolites for the detoxification enzymes mentioned previously [49,50,51,52,53].

### 3.6. Microbiological Studies

#### 3.6.1. Antimicrobial Susceptibility, MIC, and MBC

The antimicrobial susceptibility screening was performed by the cup diffusion method. The results showed that all tested *P. aeruginosa* were susceptible to the extract compared to the anti-pseudomonal activity of ciprofloxacin (2 mg/mL; Table 3). The extract displayed moderate activities against all the clinical isolates of *P. aeruginosa* and *P. aeruginosa* ATCC (12924) in the microdilution test (Table 3).

#### 3.6.2. Biofilm Formation and Quantification Assay

**Figure 4:**
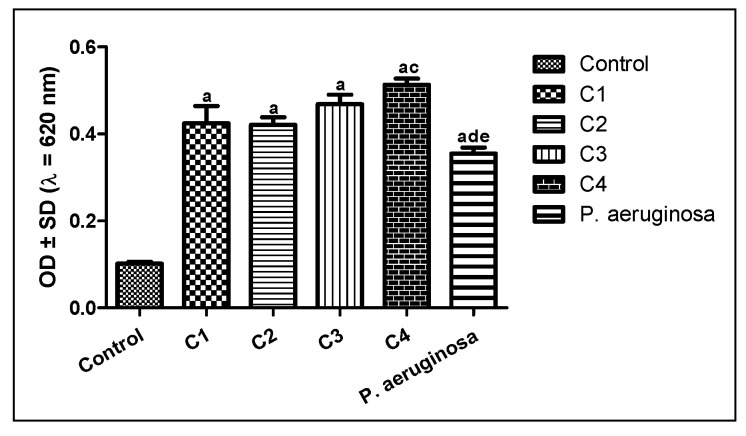
Results of biofilm formation of *P. aeruginosa*. Isolates were classified as negative (OD ≤ ODc), weak (ODc ≤ OD ≤ 2ODc), moderate (2ODc < OD ≤ 4ODc), and strong biofilm production (4ODc <OD). OD = optical density. ^a^ Significant compared to control at *p* < 0.05. ^b^ Significant compared to C1 at *p* < 0.05. ^c^ Significant compared to C2 at *p* < 0.05. ^d^ Significant compared to C3 at *p* < 0.05. ^e^ Significant compared to C4 at *p* < 0.05.

Twenty clinical isolates of *Pseudomonas* were examined for their ability to form a biofilm using the microtiter plate method. Out of the tested isolates, four bacteria showed biofilm formation, where the clinical isolate C4 exhibited the strongest biofilm formation, while C1, C2, C3, and *P. aeruginosa* ATCC (12924) showed moderate biofilm formation (Figure 4).

#### 3.6.3. Biofilm Inhibition Assay

**Figure 5:**
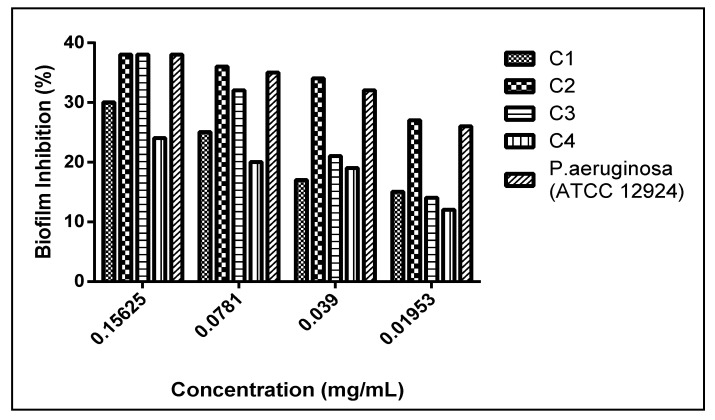
Biofilm formation inhibition activities of *L. coronopifolia* extract against *P. aeruginosa* isolates.

The biofilm inhibition activity of the extract was evaluated using three concentrations (1/2, 1/4, 1/8 MICs). The extract inhibited the biofilm formation of *P. aeruginosa* in a dose-dependent manner and ranged from 17 to 38% (Figure 5). These considerable antibiofilm properties are similar to that of polyphenol-rich extract from the bark of *Salix tetrasperma* and the leaves of *Annona glabra* and *Gynura procumbens* [54,55,56]. A recent study by Koely et al. described the antibiofilm activities of *Enydra fluctuans* against *P. aeruginosa*. They also attributed these activities to the presence of several bioactive phenolic compounds, among them kaempferol, quercetin, and luteolin and their glycosides [57]. In addition, different crude extracts from *Arbutus unedo* having high phenolic contents demonstrated comparable antibacterial activity [58].

#### 3.6.4. Synergistic Activities

**Figure 6:**
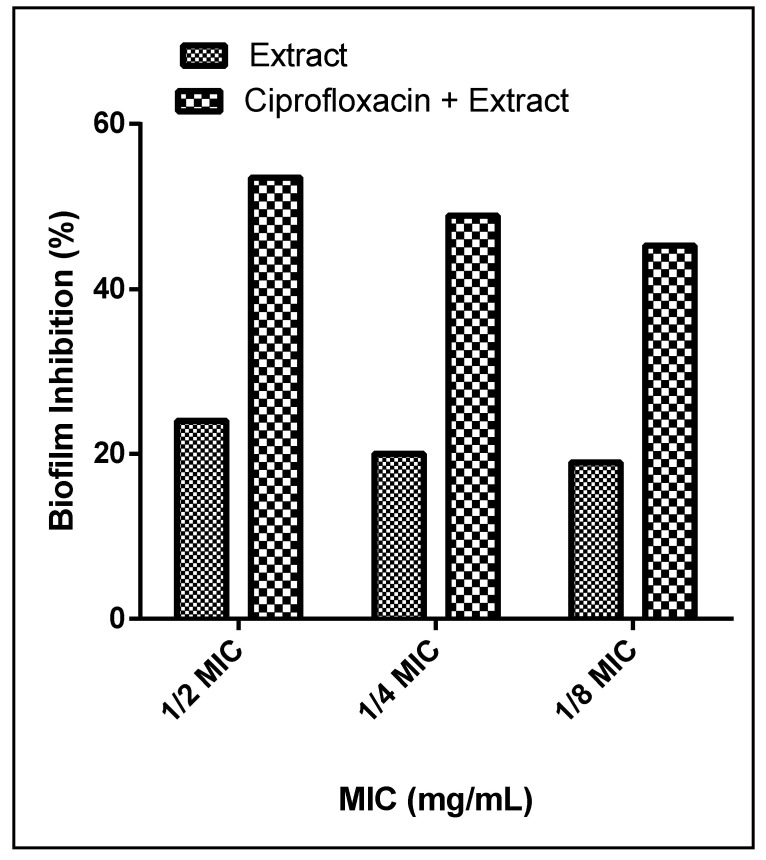
Biofilm formation activity of *L. coronopifolia* extract alone and in combination with ciprofloxacin on C4 isolate.

Antimicrobial resistance (AMR) has increased markedly in the recent years and is causing a major threat to patients’ treatment. *P. aeruginosa*, for example, has developed antibiotic resistance, and its increasing dissemination is causing severe infections in hospitals. Combinations of plant extracts with antibiotics represent a novel approach to increase their effectiveness and to overcome AMR. In an attempt to explore the synergistic activities of the extract, we combined it with the reference drug ciprofloxacin in a 1:1 ratio. The combination of ciprofloxacin and the extract substantially potentiated the reduction of biofilm from 24%, 20%, and 19% to 53.5%, 48.9%, and 45.26% at 1/2, 1/4, and 1/8 MICs, respectively (Figure 6). Our findings come in agreement with those of Okansi et al. (2013), who reported a synergy when ciprofloxacin was combined with the methanol extract of *Phyllantus muellerianus* leaves (containing flavonoids) against *P. aeruginosa* [59]. Another study described the synergistic activities against *P. aeruginosa* when zingerone extract was combined with the reference drug ciprofloxacin [60].

Coronavirus disease 2019 (COVID-19) has become the utmost and worst public health crisis of our generation. There are several risk factors associated with COVID-19, among them secondary bacterial infections, which in turn lead to serious negative outcomes and fatal clinical complications. To prevent these negative outcomes and secondary bacterial infections, patients with serious illness are treated with antibiotics. As a result, the use of antibiotics has increased, and this will significantly elevate the antibiotic resistance rates [61]. Plant extracts, with diverse secondary metabolites and several molecular targets, alone or as an adjuvant therapy, would not only boost the overall antimicrobial properties but can also work as modifying/modulating agents. This will effectively reduce the use of antibiotics and, therefore, reduce the risk of developing antibiotic resistance [62].

## 4. Conclusions

In this study, the LC-MS profiling of *L. coronopifolia* extract revealed 46 secondary metabolites. The extract displayed promising insecticidal activities against 3rd instar larvae of *C. pipiens*. The larvae showed a defensive mechanism by increasing the activities of detoxification enzymes of GSTs and α-esterases, while the toxification of *C. pipiens* was significantly observed through the reduction of CarE, AChE, and CYP450 activities. Moreover, the extract demonstrated promising antibiofilm formation against *P. aeruginosa* alone and when combined with the reference drug ciprofloxacin. To sum up, the wild plant, *L. coronopifolia,* exploits a substantial natural source to control disease carriers and manage resistant bacteria infections. Further studies are needed to evaluate the effects of *L. coronopifolia* extract on the life cycle of *C. pipiens* larvae and to explain the regulatory mechanisms of toxification.

## Figures and Tables

**Table 2:** Larvicidal activity of *L. coronopifolia* extract against the 3rd instar larvae of *Culex pipiens* 24, 48, and 72 h post treatment.

Extract (µg/mL)	24 h Post Treatment	48 h Post Treatment	72 h Post Treatment
LC_25_ (* F.l. at 95%)	20.054 (15.20–24.85)	11.274 (7.55–15.05)	8.668 (5.29–12.21)
LC_50_(* F.l. at 95%)	52.74 (44.304–62.95)	34.07 (27.43–41.41)	29.076 (22.58–36.062)
LC_95_(* F.l. at 95%)	557.50 (374.11–975.18)	505.44 (326.011–953.94)	556.28 (341.87–1151.47)
Slope ± SE	1.61 ± 0.147	1.40 ± 0.141	1.28 ± 0.138
*χ* ^2 a^	6.4544	1.9897	0.5856
Probability (P)	0.0915	0.5745	0.8997

* Fiducial limits; ^a^ chi square.

**Table 3:** Antimicrobial susceptibility, minimum inhibitory concentration (MIC), and minimal bactericidal concentration (MBC) of the *L. coronopifolia* extract and ciprofloxacin against the tested *P. aeruginosa.*

P. aeruginosa Isolates	Zone of Inhibition (mm)		Extract	
	Extract	Ciprofloxacin	MIC	MBC
			mg/mL	
C1	20	1748	0.3125	1.25
C2	20	40	0.3125	1.25
C3	20	50	0.3125	1.25
C4	28	35	0.3125	1.25
ATCC (12924)	26	22	0.1562	1.25

## Data Availability

All data are included at the manuscript.

## Sample Availability

Samples of the extract are available from the authors.
